# Editorial: Microbiome and human host interactions and their implications on human health

**DOI:** 10.3389/fmicb.2023.1135341

**Published:** 2023-02-01

**Authors:** Jazleen Leo, Chun Wie Chong, Amadeus Yeremia Pribowo, Richard Sutejo, Jonathan W. J. Lee

**Affiliations:** ^1^Yong Loo Lin School of Medicine, National University of Singapore, Singapore, Singapore; ^2^School of Pharmacy, Monash University Malaysia, Subang Jaya, Malaysia; ^3^Indonesia International Institute for Life-Sciences (i3L), Jakarta, Indonesia; ^4^Department of Gastroenterology and Hepatology, National University Health System, Singapore, Singapore; ^5^Synthetic Biology for Clinical and Technological Innovation (SynCTI), National University of Singapore, Singapore, Singapore

**Keywords:** microbiome, host interactions, human health and disease, metagenomic, mucosal microbiome

In this specialized Research Topics collection of Frontiers in Microbiology, we highlight key research papers on the microbiome and their impact on a wide variety of health conditions, such as primary hypertension, *in vitro* fertilization, axillary osmidrosis, as well as colorectal and oral squamous cell cancer. Four of the six papers in this collection provide insight into regional microbiome signatures by sampling the host site associated with the pathology directly, allowing for greater insight into the direct interactions between microbes and human host cells.

The role of gut microbes in health and disease has often been described from stool (Human Microbiome Project Consortium, 2012), which is easily sampled, rich in microbial diversity, density and abundance. Although the gut microbiome remains the most diverse microbial population in our human body, the functional heterogeneity of each body site, with its own unique microenvironment, would give rise to regional differences in the microbial populations and thus evoke different metabolic and immune functions.

The microbiota is emerging as one of the new frontiers in biomedical research. The research potential is as vast and diverse as the microorganisms housed within these communities. A large variety of microbial metabolites are produced by a balanced microbial community residing in the human body to support the homeostasis of metabolic and immune functions. Furthermore, in the event of a change in cell types and states within the same regions, unique host and environmental pressures drive mucosal microbiome heterogeneity, whereby these unique regional microbes may, in turn, influence disease propagation (Martinez-Guryn et al., 2019). Jin et al. explored the mucosal microbiome differences of colorectal cancer by sampling the microbiome of 392 patients from both colon cancer sites and adjacent healthy colon tissue. Colon cancer specimens were further subtyped as either proximal (right) or distal (left) colon to explore regional microbiome differences further. Other than identifying regional-specific microbiome signatures associated with the location of colon cancers, they were able to identify biomarkers of severity (i.e., *Fusobacteria*) and poor prognosis (Jin et al.). Similarly, Zhang et al. explored the tongue microbiome of 20 young and 20 elderly patients with oral squamous cell cancer. By using supervised machine learning to complement their differential abundance microbial analysis, they could accurately distinguish (sensitivity: 0.86, specificity: 0.94) between a young and elderly tongue cancer microbiome (Zhang et al.). It was further proposed that microbiota of the younger oral cancers were more associated with propagating inflammatory components and increased oxidative stress than the elderly. These might further lead to tumorigenesis, and thus driving a more aggressive disease course.

The following two papers by Li et al. and Wang et al. study skin and lower genital tract microbiomes, respectively, with the promise of clinical translation of microbial-based biomarkers and therapeutics to treat axillary osmidrosis and improve *in vitro* fertilization-embryo transfer. Axillary osmidrosis is a condition of offensive order resulting from the bacterial decomposition of apocrine secretions in the armpits (Morioka et al., 2020). Detrimental psychosocial effects often accompany individuals with this condition. Here, Li et al. demonstrated efficacy in the treatment of osmidrosis *via* topical application of a probiotic *L. bulgaricus* for 28 days in a pilot study of 10 patients, which was proposed to decrease the abundance of pathogenic *Corynebacterium*. In another study, Wang et al. recruited 150 patients undergoing *in vitro* fertilization-embryo transfer (IVF-ET). They were able to associate a distinct microbial signature in the cervix, consisting of an increased abundance of *Romboutsia, Anaerococcus* with success with IVF-ET, and conversely an increased abundance of *Bifidobacterium, Prevotella* associated with failure of IVF-ET (Wang et al.). These results support the potential of using lower genital microbial profiling to predict chances of pregnancy with IVF-ET, with the potential of regional probiotics used to correct localized dysbiosis of the cervix and vaginal, thereby increasing the success rates of IVF-ET.

Fungi are a key but often neglected element of the human microbiome. Boahen et al. reviewed how the overpopulation of *Candida* fungi in the vaginal microbiota can lead to vulvovaginal candidiasis. When *Candida* species monopolize the microbiota, they transition into the biofilm mode of growth, which enhances their virulence by hyphae formation and dispersion of yeast cells. *Candida* species also secrete quorum-sensing molecules (QSMs) such as farnesol and tyrosol to aid in the formation and maintenance of biofilm (Wongsuk et al., 2016). The first-in-line therapy for vulvovaginal candidiasis is an antifungal agent. However, frequent administration can lead to resistance and persister cells which may contribute to recurrent infections. Persistor *C. albicans* can form biofilms among themselves and, in some cases, a polymicrobial biofilm with neighboring bacteria and fungi to enhance protection against the fungicide. Given the critical role of biofilm formation in causing infection and developing resistance, therapeutics that can destroy *Candida* biofilms and disrupt QSM signaling are warranted. Biotics may also be used to keep *Candida* species at bay and restore microbial balance in combination with antifungals.

Zheng et al. investigated the effects of anti-hypertension medication on the gut microbiota of hypertensive patients. They found that most patients were prescribed medication that activates the adenosine monophosphate-activated protein kinase (AMPK) pathway, which is an essential upstream macrophage activator. Wild-type and AMPK-knockout hypertensive mice models were used to elucidate the alteration in the microbiome. Bacteria producing short-chain fatty acids, glycan and trimethylamine were more abundant in the primary hypertensive patients taking anti-hypertensive medication than in the healthy controls (Zheng et al.). These bacteria may play essential roles in the modulation of blood pressure. Separately, functional analysis showed that glycan biosynthesis and metabolism pathways were also enhanced in hypertensive patients. The authors speculated that the AMPK activation *via* antihypertensive medications might trigger macrophages to regulate the gut microbiota in favor of bacteria that may help regulate blood pressure. Although the exact mechanisms have yet to be elucidated, this paper presents an exciting new direction for future hypertension research.

Currently, technologies to study low-biomass samples from tissue biopsies or other regional sites are limited to 16S rRNA amplicon sequencing. As the technology progresses, we anticipate advancements in understanding microbial gene function for a holistic interpretation of microbiome-host interactions. This would further fuel the discovery and translation of targeted microbiome-based interventions for microbiome-based precision medicine.

## Author contributions

All authors listed have made a substantial, direct, and intellectual contribution to the work and approved it for publication.

## References

[B1] Human Microbiome Project Consortium (2012). Structure, function and diversity of the healthy human microbiome. Nature. 486, 207–214. 10.1038/nature11234 22699609 PMC3564958

[B2] Martinez-Guryn K. Leone V. Chang E. B. (2019). Regional diversity of the gastrointestinal microbiome. Cell Host Microbe. 26, 314–324. 10.1016/j.chom.2019.08.011 31513770 PMC6750279

[B3] Morioka D. Nomura M. Lan L. Tanaka R. Kadomatsu K. (2020). Axillary Osmidrosis. Ann. Plastic Surg. 84, 722–728. 10.1097/sap.0000000000002111 31850965

[B4] Wongsuk T. Pumeesat P. Luplertlop N. (2016). Fungal quorum sensing molecules: Role in fungal morphogenesis and pathogenicity. J. Basic Microbiol. 56, 440–447. 10.1002/jobm.201500759 26972663

